# Nutritional and immune-inflammatory scoring system for predicting outcomes in newly diagnosed diffuse large B-cell lymphoma patients

**DOI:** 10.3389/fnut.2025.1591508

**Published:** 2025-07-28

**Authors:** Yu Peng, Tingting Jiang, Shuang Chen, Xinyi Tang, Yakun Zhang, Liangmei Li, Lian Li, Longrong Ran, Xuelian Wu, Jun Li, Wei Zhang, Nanjun Li, Zailin Yang, Yao Liu

**Affiliations:** ^1^Chongqing Key Laboratory for the Mechanism and Intervention of Cancer Metastasis, Department of Hematology-Oncology, Chongqing University Cancer Hospital, Chongqing, China; ^2^School of Medicine, Chongqing University, Chongqing, China

**Keywords:** DLBCL, nutritional, immune, inflammatory, score system

## Abstract

**Background:**

Diffuse large B-cell lymphoma (DLBCL) is the most common aggressive non-Hodgkin lymphoma and often carries a poor prognosis. Current prognostic systems such as the International Prognostic Index (IPI) and National Comprehensive Cancer Network (NCCN)-IPI do not incorporate patients’ nutritional, immune, or inflammatory status, which may affect outcomes.

**Methods:**

We retrospectively analyzed 423 newly diagnosed DLBCL patients and collected 12 clinical indicators reflecting nutritional, immune, and inflammatory status. Patients were randomly divided into training and validation sets in a 7:3 ratio. A LASSO-Cox regression model was applied to identify variables for constructing the Nutritional and Immune-Inflammatory Scoring System (NII). The independent prognostic value of NII was evaluated using univariable and multivariable Cox regression analyses. Its added prognostic value was further assessed in combination with the IPI and NCCN-IPI.

**Results:**

We developed the NII, including Nutritional Risk Screening 2002 (NRS2002), Geriatric Nutritional Risk Index (GNRI), systemic immune-inflammation index (SII), lactic dehydrogenase to albumin ratio (LAR), *β*2-microglobulin (*β*2-MG), and CD8^+^ T cells. A high NII (≥ 6) effectively identifies high-risk DLBCL patients and serves as an independent prognostic factor beyond other clinical characteristic, IPI, and NCCN-IPI. DLBCL patients with a high NII (≥ 6) exhibit significantly adverse clinical features, including older age, lower frequency of the non-GCB subtype, advanced Ann Arbor stage (III/IV), poor performance status (ECOG PS ≥ 2), involvement of ≥ 2 extranodal sites, presence of B symptoms, elevated lactate dehydrogenase (LDH) levels, and classification into higher-risk groups according to IPI and NCCN-IPI. Combining NII with IPI or NCCN-IPI significantly improves the assessment of patient prognosis compared to using IPI or NCCN-IPI alone.

**Conclusion:**

The NII score, integrating readily available nutritional, immune, and inflammatory markers, enhances prognostic accuracy in DLBCL and complements conventional scoring systems. This simple tool may aid in early identification of high-risk patients and guide personalized treatment.

## 1 Introduction

Diffuse large B-cell lymphoma (DLBCL) is a highly aggressive type of non-Hodgkin’s lymphoma (NHL), accounting for approximately 30 to 40% of NHL, with heterogeneous genetic, phenotypic, and clinical features (1–3). With the advent of the rituximab era, first-line treatment with R-CHOP regimens (rituximab, cyclophosphamide, adriamycin, vincristine, and prednisone) has significantly prolonged survival in patients with DLBCL (4). However, about 40% of DLBCL patients treated with the R-CHOP regimen still develop relapsed/refractory DLBCL. These poor outcomes may be related to factors such as gene alterations, physical fitness, and immune function (5–8). Due to the heterogeneity and individual variability of DLBCL, along with numerous prognostic factors, accurately identifying high-risk DLBCL poses a challenge. Therefore, it is critical to develop an accurate risk classification system, identify high-risk patients early, and tailor treatment plans accordingly to maximize patient survival benefits.

Over the past decades, various prognostic indices integrating both tumor-intrinsic and patient clinical characteristics have been proposed. The International Prognostic Index (IPI) remains widely used for risk stratification in DLBCL but has shown reduced efficacy in the rituximab era (9, 10). Novel prognostic evaluation systems, the National Comprehensive Cancer Network (NCCN)-IPI, offers improved accuracy in prognostic evaluation and risk stratification for DLBCL patients (11). Despite these advancements, considering the heterogeneity and aggressiveness of DLBCL, current prognostic scoring systems inadequately capture the disease’s complexity. They often omit critical factors, including nutritional parameters and biomarkers, leading to inconsistencies in risk assessment. This can result in both underestimation and overestimation of prognoses, potentially influencing clinical decisions and patient outcomes. Gene expression profiling (5), immunoglobulin repertoire clonotype analysis (12), gene mutational analyses (13–15), and novel molecular subtypes (16, 17) have provided crucial predictive insights in DLBCL, enabling more accurate individual risk prediction. Unfortunately, despite their significant prognostic value, these approaches are often expensive, time-intensive, technically demanding, and occasionally lack reproducibility, limiting their adoption in clinical practice. Therefore, identifying prognostic parameters that are more accessible and cost-effective than molecular or genomic analyses is essential both for timely risk stratification and for guiding appropriate treatment strategies in DLBCL.

In recent years, increasing research has focused on the associations among patients’ nutritional status, inflammation, immune changes, and lymphoma development. DLBCL patients frequently exhibit nutritional changes at disease onset, with some showing significant weight loss and marked alterations in nutritional indicators upon admission (18–20). Several studies have indicated that nutritional assessment tools and related indicators, such as Nutritional Risk Screening 2002 (NRS2002) (21, 22), Geriatric Nutritional Risk Index (GNRI) (20, 23), and Prognostic Nutritional Index (PNI) (20, 24), can be key factors in predicting the long-term prognosis of DLBCL. Matsukawa et al. found that low GNRI or PNI in non-GCB DLBCL patients were associated with poor survival and served as independent prognostic factors beyond the NCCN-IPI (20). Moreover, the development of DLBCL is closely related to persistent chronic inflammation and immune dysfunction (25, 26). Numerous studies have highlighted the critical roles of the inflammatory response and immune function in monitoring and predicting DLBCL prognosis (27, 28). Indeed, inflammatory markers such as the systemic immune-inflammation index (SII) (28, 29), neutrophil-to-lymphocyte ratio (NLR) (30, 31), platelet-to-lymphocyte ratio (PLR) (32), lactic dehydrogenase to albumin ratio (LAR) (33), and *β*2-microglobulin (*β*2-MG) (34, 35), have been shown to correlate with the survival outcomes of DLBCL patients. Additionally, peripheral blood T lymphocyte subsets can directly reflect a patient’s immune function status. Hatake et al. discovered a lower absolute count of peripheral blood CD4^+^ lymphocytes as an independent risk factor for poor prognosis in DLBCL patients treated with R-CHOP (36). Furthermore, lower counts of CD8^+^ T cells, and CD3^+^ T cells have been strongly linked to patient outcomes and prognosis (8, 37). In summary, nutrition-related indicators, along with inflammation and immune-related peripheral blood markers, hold potential as prognostic markers for DLBCL. However, the prognostic value of these indicators remains subject to debate, and their impact on different treatment strategies has yet to be fully elucidated.

Therefore, incorporating additional clinical and laboratory biomarkers, such as inflammatory, immune, and nutrition-related indicators, into predictive model can enhance risk assessment and support personalized treatment for DLBCL patients. This study will evaluate the prognostic value of these parameters to develop a novel scoring system of DLBCL that complements and optimizes the existing NCCN-IPI and IPI scoring systems. By integrating these nutrition, inflammation, and immunity-related factors, this study aims to more precisely identify high-risk DLBCL patients, provide a more scientific basis for treatment decisions, and ultimately improve overall survival rates and treatment outcomes for patients.

## 2 Objects and methods

### 2.1 Objects

A retrospective monocentric analysis included 423 newly diagnosed DLBCL patients treated at Chongqing University Cancer Hospital between January 2020 and September 2023. Inclusion Criteria: diagnosed with DLBCL according to the Clinical Diagnosis and Treatment Guidelines (Tumor Subsection); pathologic biopsy of the patient’s diseased lymph node or extra-nodal lesion site, and the pathology department clearly diagnosed DLBCL according to the International Classification of Diseases; age ≥18 years; received at least 4 cycles of R-CHOP or other R-based regimens (e.g., R-EPOCH: rituximab, etoposide, doxorubicin, cyclophosphamide, vincristine, prednisone; R-CDOP: rituximab, cyclophosphamide, doxorubicin liposome, vindesine, and prednisone); availability of complete clinical data, including medical history, physical examination, and laboratory data (blood counts, biochemistry, T lymphocyte subset, etc.) Exclusion criteria: patients presence of other malignancies or primary central nervous system DLBCL; those combined with severe active infectious diseases; patients with combined autoimmune diseases; incomplete clinical data. Supplementary Figure S1 outlines the patient selection process and cohort allocation for model development. This research obtained approval from the Medical Ethics Committee of Chongqing University Cancer Hospital and was conducted in accordance with the Declaration of Helsinki.

### 2.2 Data collection

Relevant patient data were collected from the electronic medical record system, including the following: (1) patient’s clinical indicators: age, gender, cell of origin (COO) classification, Ann Arbor stage, IPI, NCCN-IPI, EBV infection (EBER positive), B symptoms (presence of at least one of the following: night sweats, ≥10% weight loss over 6 months, or recurrent fever temperature >38.3°C), Eastern Cooperative Oncology Group Performance Status (ECOG PS), the site of extranodal (EN) site involvement, and adverse effects after chemotherapy (infection, myelosuppression, anemia, thrombocytopenia, agranulocytosis, digestive system disease, cardiovascular reaction). (2) All peripheral blood and serum markers assessments were performed before the beginning of treatment, including complete blood count, albumin (Alb), lactate dehydrogenase (LDH), *β*2-MG, T lymphocyte subsets (e.g., B cells, CD4^+^ T cells, CD8^+^ T cells, CD3^+^ T cells, etc.). (3) Nutritional and inflammatory-related indicators, including NRS2002, GNRI, PNI, SII, NLR, PLR, and LAR. Additional data were obtained included the date of diagnosis, date of progression, date of death or date of last contact.

The NRS2002 assessment criteria include the severity of the impact of the primary disease on nutritional status (including hematologic malignancies), recent weight changes (1–3 months), changes in food intake within 1 week, or BMI score, and over 70 years, NRS2002 score ≥3 was defined as a risk of malnutrition (38). Relevant indicators were defined as follows: GNRI = 1.489 × Alb (g/L) + 41.7 × [admission weight (kg)/ideal weight (kg)]; PNI = Alb (g/L) + 5 × lymphocyte count (10^9^/L); SII = platelet count × neutrophil count/lymphocyte count; NLR = neutrophil count/lymphocyte count; PLR = platelet count/lymphocyte count; LAR = LDH/Alb.

### 2.3 Detection methods

The complete blood count was measured with an automated hematology analyzer (XN-9000; Sysmex, Kobe, Japan). Serum Alb, LDH, and *β*2-MG levels were assessed with a Hitachi automatic analyzer (7600-010; Hitachi High-Technologies Corporation, Tokyo, Japan). T lymphocyte subsets were analyzed using flow cytometer. The absolute numbers of CD4^+^ cells, CD8^+^ cells, B cells, and CD3^+^ T cells were determined using Reagent Kit (Beijing Quantobio Biotechnology, Beijing, China) according to the manufacturer’s instructions. In brief, 50 μL of whole blood was labeled with an antibody mixture, added to a tube containing fluorescent beads, and incubated for 15 min at room temperature in the dark. After adding 450 μL of lysing solution, the samples were measured with EasyCell automatic flow cytometer (Wellgrom, Shenzhen, China). Data were analyzed using Diva software (BD Biosciences, Franklin Lakes, NJ, United States).

### 2.4 Response assessment

18F-fluorodexyglucose positron emission tomography/computed tomography (PET/CT) or computed tomography (CT) were performed for radiological evaluation. Treatment response was assessed according to the Lugano 2014 criteria (39), which incorporate both anatomical and metabolic imaging. The response categories included complete response (CR), partial response (PR), stable disease (SD), and progressive disease (PD). For patients undergoing PET/CT, metabolic response was evaluated using the Deauville 5-point scale. A Deauville score of 1–3 was considered a complete metabolic response, while scores of 4 or 5 indicated residual disease, with PR defined as a decrease in uptake relative to baseline. PD was defined by the appearance of new FDG-avid lesions consistent with lymphoma or an increase in uptake of existing lesions. For patients assessed by CT alone, CR was defined as the disappearance of all evidence of disease, including all lymph nodes regressing to ≤1.5 cm in the longest diameter. PR was defined as at least a 50% decrease in the sum of the product of diameters of up to six of the largest dominant nodes or nodal masses. PD was defined as the appearance of new lesions >1.5 cm or a ≥ 50% increase in the longest diameter of previously identified lesions. SD was defined as not meeting the criteria for CR, PR, or PD.

### 2.5 Follow-up and end points

The treatment response was evaluated according to the International Workshop criteria (40). Progression-free survival (PFS) was defined as the time interval from diagnosis to disease progression or relapse. Overall survival (OS) was defined as the time interval from diagnosis to death or the last follow-up.

### 2.6 Statistical analysis

Statistical analyses were performed using R software (version. 4.3.1). All eligible patients were randomly divided into training set and validation set at a ratio of 7:3. Missing values were imputed using the MICE method with random forest as the imputation model, implemented via the R package *mice*. The dataset with the most stable PCA structure was selected for analysis. Optimal cutoff values were determined using maximally selected rank statistics implemented in the *surv_cutpoint()* function from the R package *survminer*, based on survival outcome separation. Notably, the cutoff values of the variables were consistent for both PFS and OS. Categorical variables were analyzed using the Chi-square test or Fisher’s exact test, with results described as frequencies and percentages. The R package *survminer* was used to determine the optimal cut-off value of indicators. To avoid potential multicollinearity, we employed the least absolute shrinkage and selection operator (LASSO) Cox regression model with the R package *glmnet* to identify the most influential factors among the 12 nutritional and immune-inflammatory indicators. Scores were assigned based on the unpenalized coefficients to construct the new scoring system, the Nutritional and Immune-Inflammatory Scoring System (NII). Model performance was evaluated using the R package *caret* and *pROC*. Univariable and multivariable Cox regression analyses of the new scoring system and other clinical indicators were performed using the R package *survmine*. Indicators with a *p*-value <0.2 in the univariate Cox regression analysis (log-rank test) were included in the multivariate Cox regression analysis (41, 42). Results were presented as hazard ratio (HR), 95% confidence interval (CI), and *p-*values. The Kaplan–Meier method was used to plot survival curves with package in R, and differences between Kaplan–Meier curves for PFS and OS were assessed using the log-rank test. The level of significance was set at *p*-value <0.05.

## 3 Result

### 3.1 Baseline characteristics

The baseline patient characteristics are presented in Table 1. 179 patients (42.32%) were older than 60 years. A total of 242 patients (57.21%) were male. According to the COO classification, 122 patients (28.84%) were non-GCB and 301 (71.16%) were GCB. Ann Arbor stage (stage III/IV) was noted in 269 patients (63.59%). 131 patients (89.1%) exhibited worse performance status (ECOG PS ≥ 2). Optimal cutoff values for nutritional and immune-inflammatory indicators (Supplementary Table S1; Supplementary Figure S2) were determined, and baseline characteristics were comparable between the training and validation cohorts (Table 1). Of these, a threshold of 3 for NRS2002 and 98 for GNRI were applied in this analysis (38, 43, 44), based on previously published studies. Additionally, bulky disease was defined as a tumor diameter ≥7.5 cm, in line with the RICOVER-60 trial criteria (45, 46). The LDH threshold of 250 U/L was the upper limit of normal in our laboratory (9).

**Table 1:** Patients’ baseline clinical and demographic characteristics.

Characteristics	Total (*N* = 423), *n* (%)	Training set (*N* = 296), *n* (%)	Validation set (*N* = 127), *n* (%)	*p-*value
Age, >60 years	179 (42.32)	128 (43.24)	51 (40.16)	0.630
Gender, Male	242 (57.21)	172 (58.11)	70 (55.12)	0.644
COO classification, non-GCB	122 (28.84)	86 (29.05)	36 (28.35)	0.976
Ann Arbor stage, III/IV	269 (63.59)	191 (64.53)	78 (61.42)	0.618
ECOG PS, ≥2	131 (30.96)	88 (29.73)	43 (33.86)	0.467
Tumor size, ≥7.5 cm	55 (13.00)	40 (13.51)	15 (11.81)	0.749
EN site involvement, ≥2 EN site	137 (32.38)	92 (31.08)	45 (35.43)	0.445
EBV infection, yes	24 (5.67)	16 (5.41)	8 (6.30)	0.893
B symptoms, yes	112 (26.48)	77 (26.01)	35 (27.56)	0.834
LDH, >250 U/L	221 (52.25)	152 (51.35)	69 (54.33)	0.648
NRS2002, ≥3	89 (21.04)	62 (20.95)	27 (21.26)	1.000
GNRI, ≤98	150 (35.46)	106 (35.81)	44 (34.65)	0.905
PNI, ≥45.85	179 (42.32)	126 (42.57)	53 (41.73)	0.959
SII, ≥402.71	310 (73.28)	216 (72.97)	94 (74.02)	0.919
NLR, ≥3.76	160 (37.83)	116 (39.19)	44 (34.65)	0.439
PLR, ≥153.3	256 (60.52)	179 (60.47)	77 (60.63)	1.000
LAR, ≥158.52	277 (65.48)	197 (66.55)	80 (62.99)	0.552
*β*2-MG, ≥4.75 mg/L	95 (22.46)	63 (21.28)	32 (25.20)	0.449
B cells, <116 cells/μL	203 (48.00)	148 (50.00)	55 (43.31)	0.247
CD4^+^ T cells, <575 cells/μL	275 (65.01)	188 (63.51)	87 (68.50)	0.381
CD8^+^ T cells, <222 cells/μL	122 (28.84)	87 (29.39)	35 (27.56)	0.792
CD3^+^ T cells, <857 cells/μL	193 (45.63)	134 (45.27)	59 (46.46)	0.906
IPI				0.707
Low	142 (33.57)	104 (35.14)	38 (29.92)	
Low-intermediate	87 (20.57)	58 (19.59)	29 (22.84)	
High-intermediate	106 (25.06)	72 (24.32)	34 (26.77)	
High	88 (20.80)	62 (20.95)	26 (20.47)	
NCCN-IPI				0.930
Low	65 (15.37)	45 (15.20)	20 (15.75)	
Low-intermediate	161 (38.06)	112 (37.84)	49 (38.58)	
High-intermediate	155 (36.64)	111 (37.50)	44 (34.65)	
High	42 (9.93)	28 (9.46)	14 (11.02)	
Treatment regimen				0.379
R-CHOP	255 (60.28)	183 (61.82)	72 (56.69)	
Other R regimens	168 (39.72)	113 (38.18)	55 (43.31)	
Treatment cycles				0.974
4–5	72 (17.02)	51 (17.23)	21 (16.54)	
≥6	351 (82.98)	245 (82.77)	106 (83.46)	

COO, the cell of origin; ECOG PS, Eastern Cooperative Oncology Group Performance Status; EN, extranodal; LDH, lactate dehydrogenase; NRS2002, Nutritional Risk Screening 2002; GNRI, Geriatric Nutritional Risk Index; PNI, Prognostic Nutritional Index; SII, systemic immune-inflammation index; NLR, neutrophil-to-lymphocyte ratio; PLR, platelet-to-lymphocyte ratio; LAR, lactic dehydrogenase to albumin ratio; *β*2-MG, *β*2-microglobulin; IPI, International Prognostic Index; NCCN-IPI, National Comprehensive Cancer Network International Prognostic Index; R-CHOP, rituximab, cyclophosphamide, adriamycin, vincristine, and prednisone; other R regimens, R-EPOCH (rituximab, etoposide, doxorubicin, cyclophosphamide, vincristine, prednisone), R-CDOP (rituximab, cyclophosphamide, doxorubicin liposome, vindesine, and prednisone), etc.

### 3.2 Developing the NII

**Figure 1:**
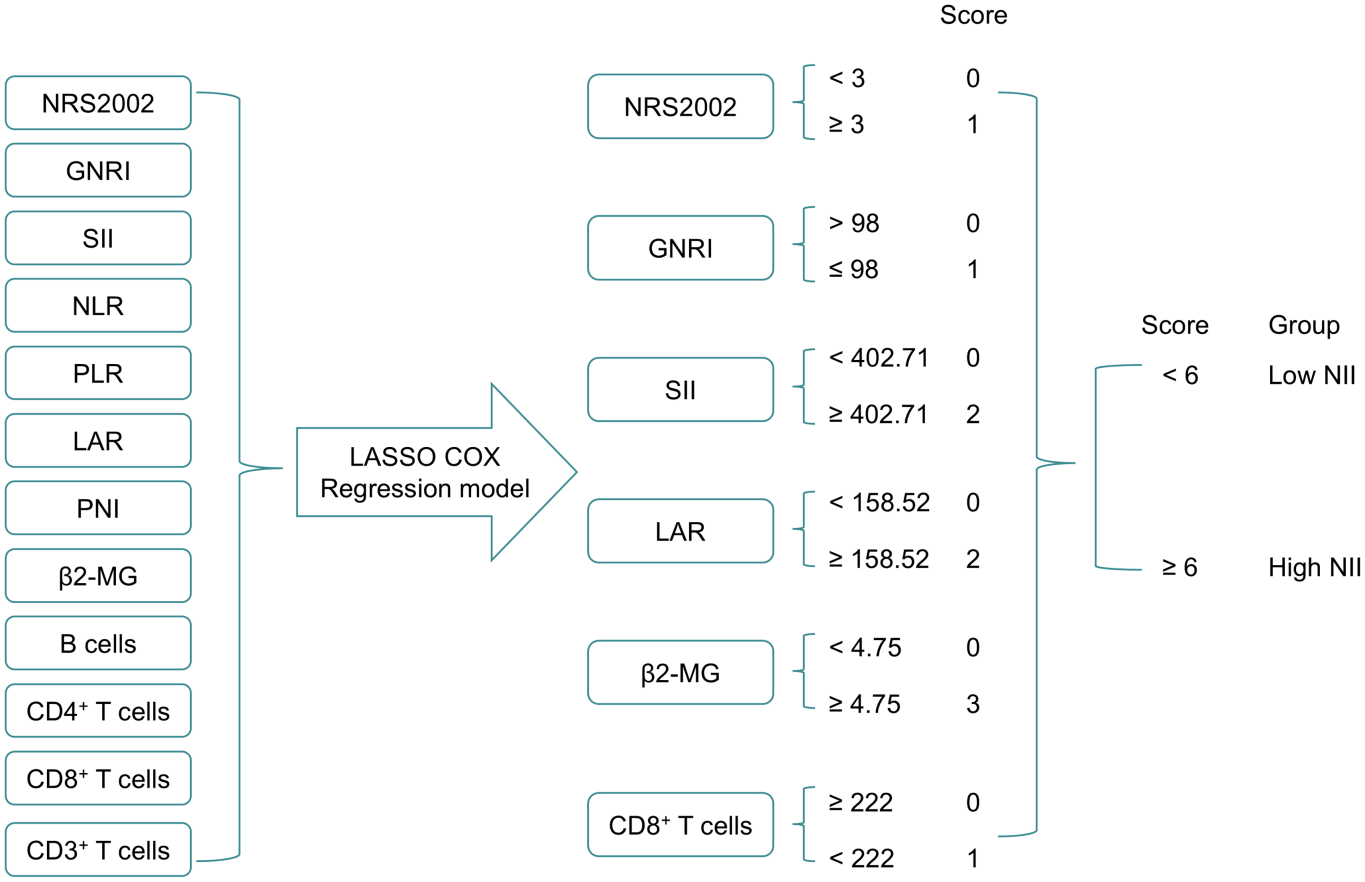
The process diagram of NII construction and risk stratification. NRS2002, Nutritional Risk Screening 2002; GNRI, Geriatric Nutritional Risk Index; PNI, Prognostic Nutritional Index; SII, systemic immune-inflammation index; NLR, neutrophil-to-lymphocyte ratio; PLR, platelet-to-lymphocyte ratio; LAR, lactic dehydrogenase to albumin ratio; *β*2-MG, *β*2-microglobulin; NII, nutritional and immune-inflammatory scoring system.

Figure 1 illustrates the process of constructing the NII. 12 nutritional and immune-inflammatory indicators were evaluated via LASSO Cox regression analysis (Supplementary Figure S3), and the six most valuable prognostic variables were selected to develop the NII. These included NRS2002, GNRI, SII, LAR, *β*2-MG, and CD8^+^ T cells, all derived from routine peripheral blood indicators. Subsequently, these variables were then entered into an unpenalized multivariate Cox regression model, and each was assigning scores based on its *β*-coefficient (Supplementary Table S2). High *β*2-MG level (≥4.75 mg/L) were scored as 3, while other values were scored as 0. High SII (≥402.71) and high LAR level (≥158.52) was scored as 2, while low level was scored as 0. High NRS2002 (≥3), low GNRI (≤98), low CD8^+^ T cells (<222 cells/μL) were each scored as 1, while other levels were scored as 0. The NII was calculated by summing the scores for each indicator. Based on the median of NII, a cutoff of NII ≥ 6 was established.

### 3.3 Assessing the performance of NII

**Figure 2:**
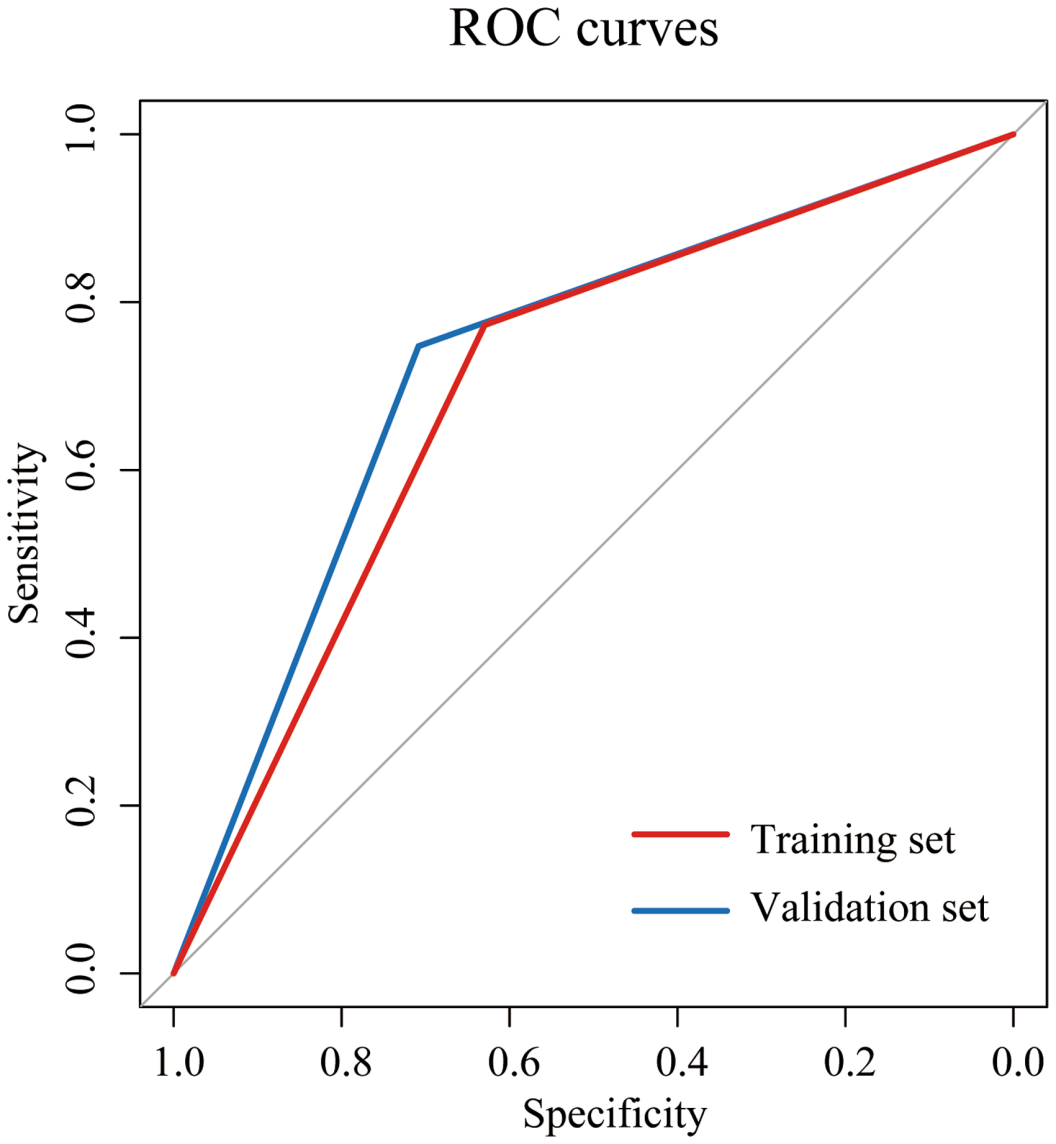
Receiver operating characteristic (ROC) curves for overall survival (OS) of NII in training set (red) and validation set (blue).

**Figure 3:**
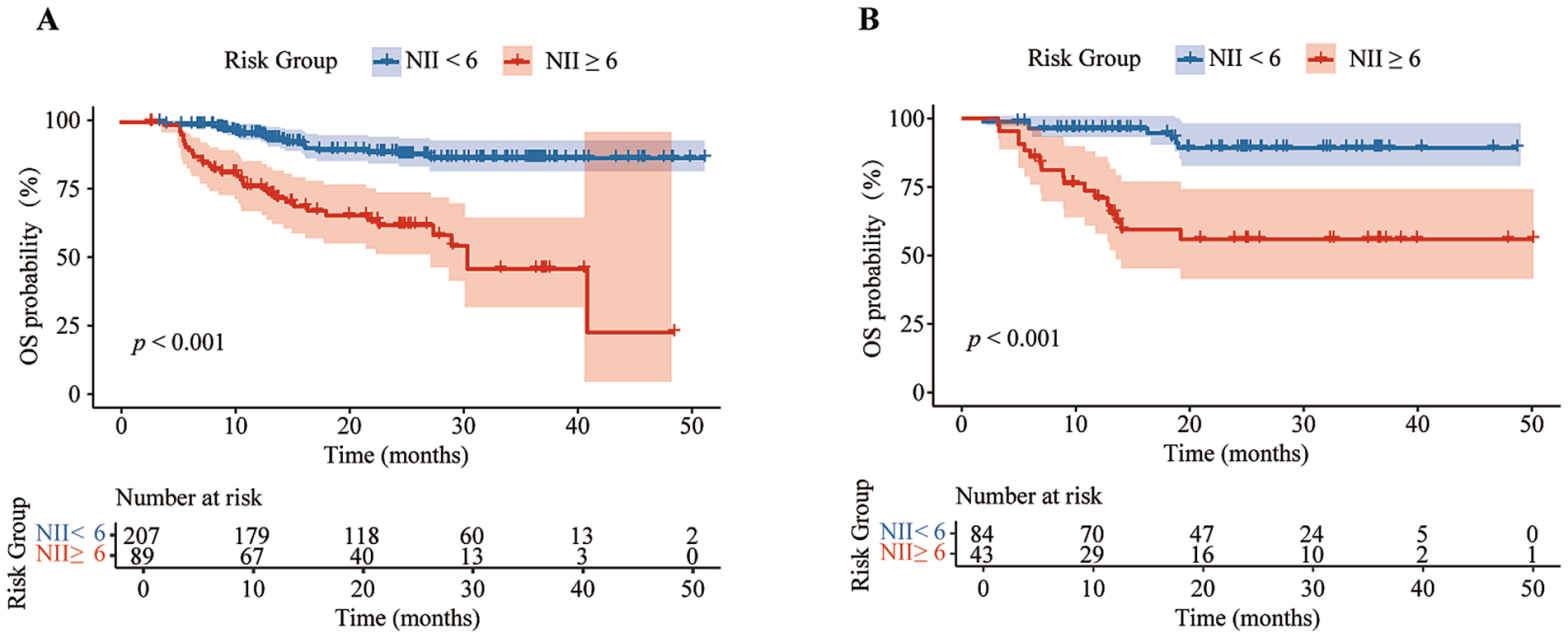
Kaplan–Meier analysis of overall survival (OS) curves between different NII groups in training set **(A)** and validation set **(B)**.

Based on the NII, we categorized the patients into high NII group (≥6) and low NII group (<6), and constructed a Cox proportional hazards model to predict the OS of the patients. The model exhibited satisfactory performance in the validation set, with a sensitivity of 0.708, specificity of 0.748, accuracy of 0.740, precision of 0.395, F1 score of 0.507, and an area under the curve (AUC) of 0.728 (Table 2). These results are similar to those observed in the training set. The receiver operating characteristic (ROC) curves for both the training and validation sets were closely aligned (Figure 2). Further analysis of OS in the high NII (≥6) and low NII (<6) groups (Figure 3) revealed that the OS probability of the high NII group was significantly lower than that of the low NII group in both the training and validation sets (*p* < 0.001).

**Table 2:** Comparison of the predictive performance of NII in training and validation sets.

Group	Performance index						
	Sensitivity	Specificity	Accuracy	Precision	F1 score	AUC	*p-*value
Training set	0.630	0.773	0.747	0.382	0.475	0.701	<0.0001
Validation set	0.708	0.748	0.740	0.395	0.507	0.728	<0.0001

AUC, area under the curve.

### 3.4 NII identified as an independent risk factor in DLBCL patients

**Figure 4:**
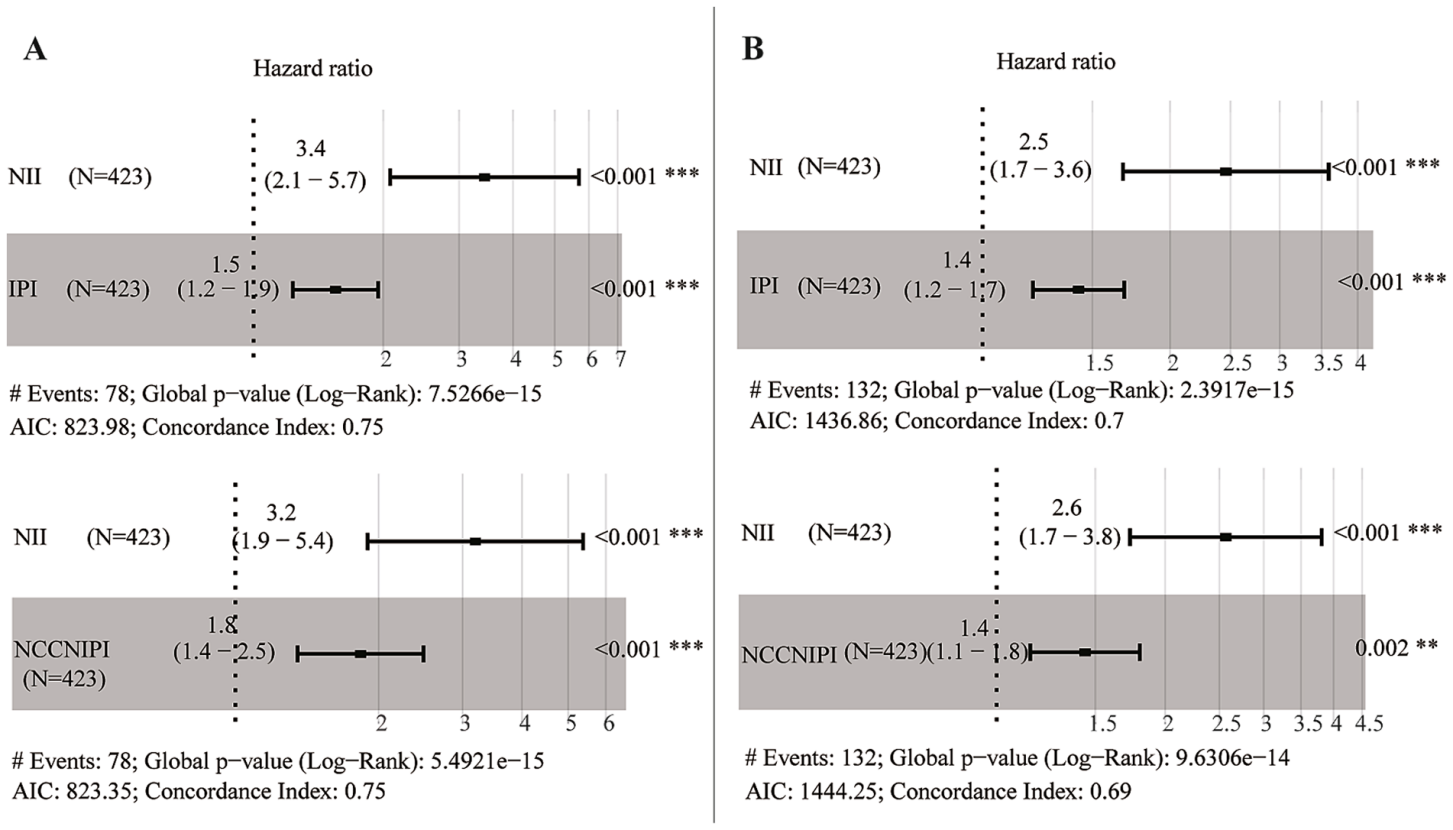
A multivariable Cox proportional hazards. Analysis for OS **(A)** and PFS **(B)** incorporating NII and IPI/NCCN-IPI.

Univariable Cox regression analysis revealed that NII (<6 vs. ≥ 6), age (>60 vs. ≤ 60), Ann Arbor stage (III/IV vs. I/II), ECOG PS (≥2 vs. < 2), EN site involvement (≥2 EN site vs. < 2 EN site), and LDH (>250 U/L vs. ≤ 250 U/L) were significant factors associated with OS and PFS in DLBCL patients (*p* < 0.01) (Table 3; Supplementary Table S3). In multivariable Cox regression analysis for OS in DLBCL patients, NII (<6 vs. ≥ 6, HR, 3.22; 95% CI, 1.90–5.44; *p* < 0.001), and age (>60 vs. ≤ 60, HR, 2.20; 95% CI, 1.35–3.58; *p* = 0.002), were the independent risk factors (Table 3). Similar results were also observed in the multivariable Cox analysis for PFS (Supplementary Table S3). These results establish NII as an independent risk factor for OS. Furthermore, multivariable Cox proportional hazards analysis confirmed that NII remained an independent prognostic factor, distinct from IPI and NCCN-IPI (Figure 4).

**Table 3:** The univariate and multivariate analysis for overall survival in DLBCL patients.

Characteristics	Univariable analysis		Multivariable analysis	
	HR (95% CI)	*p-*value	HR (95% CI)	*p-*value
NII (<6 vs. ≥ 6)	5.11 (3.20–8.16)	<0.001	3.22 (1.90–5.44)	<0.001
Gender (Male vs. Female)	1.19 (0.75–1.87)	0.457		
Age (>60 vs. ≤ 60)	2.96 (1.86–4.73)	<0.001	2.20 (1.35–3.58)	0.002
COO classification (non-GCB vs. GCB)	0.81 (0.48–1.35)	0.416		
Ann Arbor stage (III/IV vs. I/II)	3.09 (1.70–5.61)	<0.001	1.66 (0.83–3.34)	0.154
ECOG PS (≥2 vs. < 2)	1.99 (1.26–3.12)	0.002	1.45 (0.91–2.32)	0.120
Tumor size (≥7.5 cm vs. < 7.5 cm)	1.09 (0.57–2.06)	0.801		
EN involvement (≥2 EN site vs. < 2 EN site)	1.85 (1.19–2.89)	0.007	1.27 (0.78–2.09)	0.338
EBV infection (Yes vs. No)	0.63 (0.20–2.00)	0.432		
B symptoms (Yes vs. No)	1.23 (0.76–1.98)	0.408		
LDH (>250 U/L vs. ≤ 250 U/L)	2.45 (1.51–3.99)	<0.001	1.07 (0.61–1.88)	0.815
Treatment regimen (R-CHOP vs. other R regimens)	1.06 (0.68–1.66)	0.805		
Treatment cycles (4–5 vs. ≥ 6)	1.69 (1.00–2.86)	0.051	1.36 (0.79–2.34)	0.267

NII, nutritional and immune-inflammatory scoring system; COO, the cell of origin; ECOG PS, Eastern Cooperative Oncology Group Performance Status; EN, extranodal; LDH, lactate dehydrogenase; R-CHOP, rituximab, cyclophosphamide, adriamycin, vincristine, and prednisone; other R regimen, R-EPOCH (rituximab, etoposide, doxorubicin, cyclophosphamide, vincristine, prednisone), R-CDOP (rituximab, cyclophosphamide, doxorubicin liposome, vindesine, and prednisone), etc.

### 3.5 Compared clinical characteristics of patients with low NII and high NII

Compared to the low NII group (<6), the high NII group (≥6) demonstrated significantly worse clinical features, including advanced age (>60 years, 58.33%), higher Ann Arbor stage (III/IV, 87.12%), lower frequency of the non-GCB subtype (20.45%), poor performance status (ECOG PS ≥ 2, 43.94%), involvement of ≥2 EN sites (44.70%), presence of B symptoms (38.64%), elevated LDH level (>250 U/L, 78.03%), and higher risk stratification in both IPI (high-intermediate: 29.55%; high: 43.18%) and NCCN-IPI (high-intermediate: 50.76%; high: 26.52%) (Table 4). Additionally, compared to the low NII group, the high NII group exhibited poorer treatment response and increased treatment-related toxicities, including infection, myelosuppression, anemia, and agranulocytosis (Table 5).

**Table 4:** Baseline characteristics of DLBCL patients according to NII.

Characteristics	Low NII (<6), *n* (%)	High NII (≥6), *n* (%)	*p-*value
Age, >60 years	102 (35.05)	77 (58.33)	<0.001
Gender, Male	157 (53.95)	85 (64.39)	0.057
COO (Hans), non-GCB	95 (32.65)	27 (20.45)	0.014
Ann Arbor stage, III/IV	154 (52.92)	115 (87.12)	<0.001
ECOG PS, ≥2	73 (25.09)	58 (43.94)	<0.001
Tumor size, ≥7.5 cm	35 (12.03)	20 (15.15)	0.466
EN site involvement, ≥2 EN site	78 (26.80)	59 (44.70)	<0.001
EBV infection, yes	15 (5.15)	9 (6.82)	0.647
B symptoms, yes	61 (20.96)	51 (38.64)	<0.001
LDH, >250 U/L	118 (40.55)	103 (78.03)	<0.001
IPI			<0.001
Low	128 (43.99)	14 (10.61)	
Low-intermediate	65 (22.34)	22 (16.67)	
High-intermediate	67 (23.02)	39 (29.55)	
High	31 (10.65)	57 (43.18)	
NCCN-IPI			<0.001
Low	61 (20.96)	4 (3.03)	
Low-intermediate	135 (46.39)	26 (19.70)	
High-intermediate	88 (30.24)	67 (50.76)	
High	7 (2.41)	35 (26.52)	

COO classification, the cell of origin classification; ECOG PS, Eastern Cooperative Oncology Group Performance Status; EN site involvement, extranodal site involvement; LDH, lactic dehydrogenase; IPI, International Prognostic Index; NCCN-IPI, National Comprehensive Cancer Network IPI.

**Table 5:** Response and adverse events of treatment between low NII and high NII.

Treatment-related variables	Low NII (≤6), *n* (%)	High NII (>6), *n* (%)	*p-*value
Treatment response			<0.001
CR + PR	263 (90.38)	99 (75.00)	
SD + PD	12 (4.12)	21 (15.91)	
NA	16 (5.50)	12 (9.09)	
Treatment related toxicity			
Infection	96 (32.99)	64 (48.48)	0.003
Myelosuppression	146 (50.17)	89 (67.42)	0.001
Anemia	85 (29.21)	58 (43.94)	0.004
Thrombocytopenia	35 (12.03)	20 (15.15)	0.466
Agranulocytosis	77 (26.46)	51 (38.64)	0.016
Digestive system disease	28 (9.62)	14 (10.61)	0.890
Cardiovascular reaction	12 (4.12)	10 (7.58)	0.213

CR, complete response; PR, partial response; SD, stable disease; PD, progressive disease; NA, not available.

### 3.6 Prognostic value of NII combined with IPI or NCCN-IPI for survival in DLBCL patients

**Figure 5:**
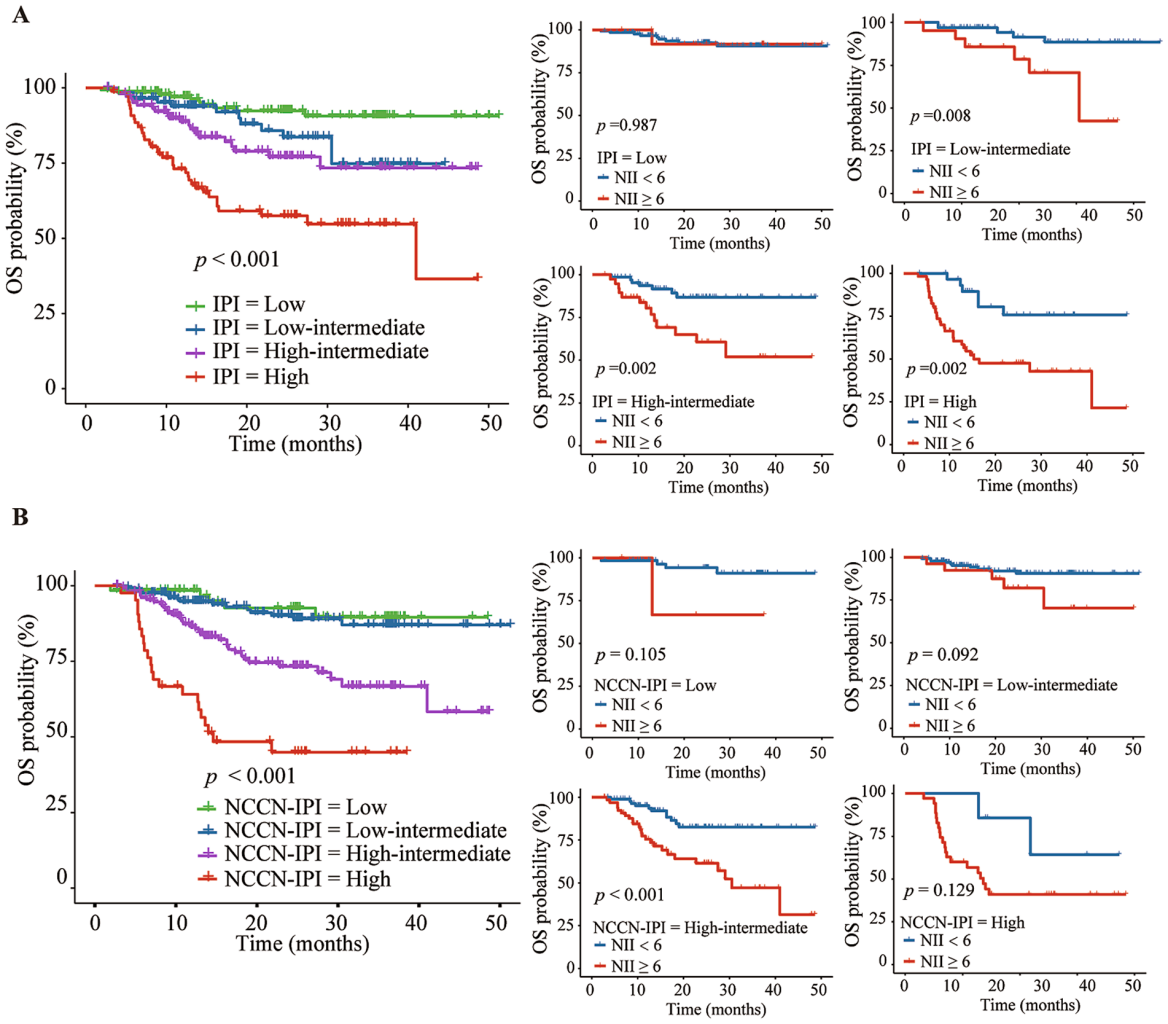
Kaplan–Meier analysis of NII combined with IPI **(A)** or NCCN-IPI **(B)** for predicting the overall survival (OS) of DLBCL patients.

**Figure 6:**
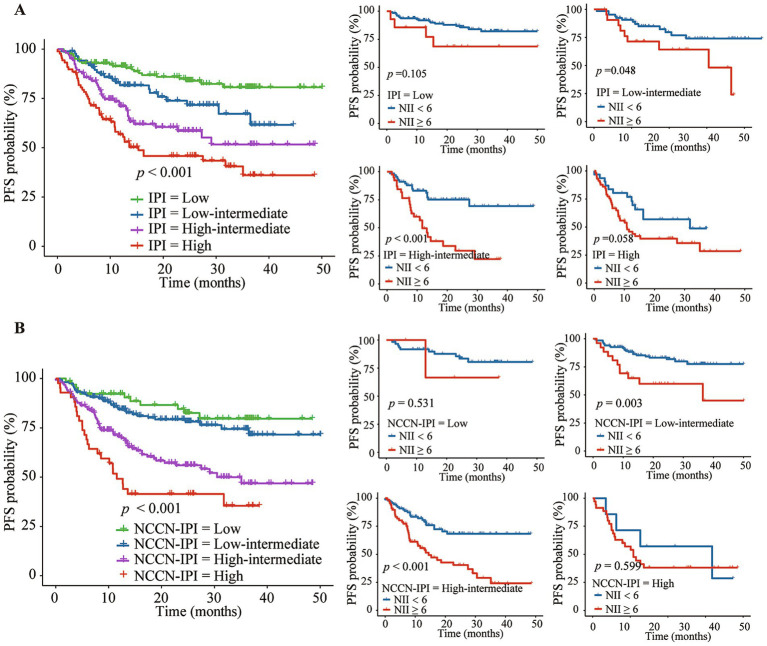
Kaplan–Meier analysis of NII combined with IPI **(A)** or NCCN-IPI **(B)** for predicting the progression-free survival (PFS) of DLBCL patients.

The Kaplan–Meier analysis demonstrated that a high NII score was associated with poorer OS and PFS across three IPI risk groups: low-intermediate group (the 3-year OS of 42.4% vs. 88.5%, *p* = 0.007; the 3-year PFS of 48.2% vs. 74.1%, *p* = 0.048), high-intermediate group (the 3-year OS of 51.9% vs. 86.8%, *p* = 0.002; the 3-year PFS of 22.2% vs. 69.4%, *p* < 0.001), and high group (the 3-year OS of 42.8% vs. 75.7%, *p* = 0.002; the 3-year PFS of 28.6% vs. 48.7%, *p* = 0.058) (Figures 5A, 6A). Further analysis of the outcomes of patients within the four NCCN IPI risk levels found that a high NII score was significantly associated with poorer OS and PFS in the low-intermediate (the 3-year OS of 70.3% vs. 90.6%, *p* = 0.092; the 3-year PFS of 59.9% vs. 77.5%, *p* = 0.002) and high-intermediate groups (the 3-year OS of 47.1% vs. 82.6%, *p* < 0.001; the 3-year PFS of 24.1% vs. 68.2%, *p* < 0.001) (Figures 5B, 6B).

## 4 Discussion

Over the past few decades, prognostic stratification systems of DLBCL, such as IPI, aaIPI, and NCCN-IPI, have shown certain limitations in effectively identifying high-risk patients. These survival scoring systems primarily focus on clinical characteristics, overlooking the impact of factors such as nutritional status, inflammatory response, and changes in immune function on prognosis. This oversight may result in certain high-risk patients not being accurately identified, ultimately impacting their treatment and outcomes. To address these shortcomings, we have developed NII that evaluates patient nutrition, immune status, and inflammation using easily accessible peripheral blood indicators, aiming to more accurately stratify the risk for DLBCL patients.

DLBCL is a highly aggressive tumor characterized by rapid progression and systemic inflammatory response, which can lead to symptoms such as decreased appetite, weight changes, and muscle loss, ultimately affecting in the nutritional status of patients. Nutritional statuses could be associated with worse prognosis possibly by leading to immunosuppression, delayed wound healing, and negative effects on the cardiopulmonary function (47, 48). In this study, we incorporated nutritional indicators, including NRS2002 and GNRI, into NII. NRS2002 is a widely used clinical tool for assessing nutritional risk, particularly in preoperative evaluation of hospitalized patients. Patients with an NRS2002 score of ≥3 are classified as malnourished and are typically associated with a poorer prognosis (21). GNRI is a more reliable predictor of prognosis in hematologic malignancies compared to serum Alb levels, body weight, or BMI alone (20, 23). A lower GNRI is linked to worse short- and long-term outcomes for DLBCL patients and is associated with adverse clinical features, including ECOG PS ≥ 2, Ann Arbor stage III-IV, B symptoms, and extranodal disease (49, 50). Malnourished patients are more prone to treatment-related toxicities and early treatment discontinuation, ultimately leading to worse clinical outcomes (51, 52). Studies have demonstrated that nutritional interventions, including personalized dietary plans and supplementation, can mitigate treatment-related adverse effects and enhance both treatment efficacy and survival outcomes in cancer patients (53). These findings underscore the correlation between poor nutritional status and worse prognosis in DLBCL patients.

Poor nutritional status in DLBCL is closely associated with systemic inflammation and tumor burden (20), with inflammation potentially worsening nutritional status through the loss of adipose tissue, skeletal muscle, and appetite (54). NII included inflammatory indicators (SII, LAR, *β*2-MG), which were calculated from complete blood count and biochemical parameters. Numerous studies have demonstrated the significant prognostic value of these inflammatory indicators in DLBCL (28, 31–33, 55). Hu et al. developed SII, a novel inflammatory indicator that accurately reflects both local immune response and systemic inflammatory status (56). SII is an immune-inflammatory biomarker based on platelet, neutrophil, and lymphocyte counts. Increased neutrophils and platelets lead to the release of various inflammatory factors and cytokines, promoting angiogenesis and tumor invasion, while lymphocytes play a role in inhibiting tumor cell growth and metastasis (28). Therefore, elevated SII levels are associated with poorer prognosis in DLBCL patients (28). In certain clinical practices, SII has demonstrated greater accuracy in reflecting inflammatory status and tumor activity compared to PLR and NLR (29, 55). Additionally, a meta-analysis found that higher SII is closely associated with the presence of B symptoms, Ann Arbor stage III-IV, high-intermediate/high NCCN-IPI, as well as increased NLR and PLR in DLBCL patients (28). Elevated SII also predicts poorer outcomes and increased likelihood of disease progression in DLBCL patients (28). LAR, the ratio of LDH to Alb, plays an important role in the assessing immune status of various cancers (57). LDH level reflects tumor growth, invasive potential, and immunosuppression (58), while serum Alb levels indicate nutritional status. The combination of these two markers provides greater predictive value for the prognosis of tumor patients (59). In our study, LAR was also included in NII, indicating that LAR can effectively predict the prognosis of DLBCL patients. Studies have shown that elevated *β*2-MG levels are an independent prognostic risk factor for DLBCL patients in the rituximab era, particularly useful for identifying high-risk advanced-stage patients (34, 35, 60–62). Our findings are consistent with previous reports, demonstrating that elevated *β*2-MG levels are linked to poorer OS in patients. This may be due to *β*2-MG’s role in promoting tumor self-renewal, aiding apoptosis evasion, and facilitating bone metastasis, thus worsening prognosis in DLBCL patients (63–65).

The peripheral immune system, a crucial defense mechanism, significantly impacts the diagnosis and prognosis of DLBCL. Delfau-Larue et al. observed frequent deficiencies in CD4^+^ T cells and CD8^+^ T cells in DLBCL patients at diagnosis (66). Compared to healthy individuals, DLBCL patients have lower levels of CD3^+^ T cells, CD4^+^ T cells, and CD8^+^ T cells, with these counts decreasing as the IPI score increases (8, 67). Circulating CD3^+^ T cells, CD4^+^ T cells, and CD8^+^ T cells have been previously reported as predictive or prognostic biomarkers in hematological diseases (7, 36, 68–70). Moreover, age-related shifts within CD4^+^ and CD8^+^ T-cell subpopulations may influence total counts without necessarily indicating improved anti-tumor immunity (71, 72). Due to the retrospective nature of this study, detailed immunophenotyping data on CD4^+^ and CD8^+^ subpopulations were unavailable, limiting further subset analyses. In this study, CD8^+^ T cells were incorporated into NII, highlighting its prognostic significance in DLBCL. These findings suggest a complex relationship between lymphocyte cell subsets and DLBCL prognosis, highlighting the need for further research into the underlying mechanisms.

Previous studies have emphasized the significance of patient nutrition status, inflammatory response, and immune function in the survival of DLBCL patients (28, 31, 32, 49, 55). However, single indicators fail to comprehensively reflect the overall nutritional and immune-inflammatory status of individuals. Some studies integrate highly correlated nutritional, inflammatory, or immune markers into multivariable Cox regression models to identify independent prognostic factors, which can present statistical issues. To address multicollinearity, we utilized the LASSO Cox regression model for effective indicators screening. Ultimately, two nutritional indicators (NRS2002, GNRI) and four immune-inflammatory markers (SII, LAR, *β*2-MG, CD8^+^ cells) were selected. Further, coefficients were assigned to develop NII based on the multivariable Cox regression analysis, which effectively predicts the prognosis of DLBCL patients independently of IPI and NCCN-IPI. Patients with high NII exhibit characteristics such as age (>60 years), male, B symptoms, GCB subtype, Ann Arbor stage III-IV, ECOG PS ≥ 2, EN site involvement ≥2 sites, elevated LDH, and high-intermediate/high IPI and NCCN-IPI. This study found that the combined NII enhances prognostic assessment in DLBCL patients compared to IPI or NCCN-IPI alone, improving the identification of those with poorer outcomes. The indicators included in this study are based on readily accessible peripheral blood, offering a widely used method with non-invasive, simple accessibility. Furthermore, compared to other prediction models such as nomograms, random forests, this scoring system simplifies model application and can be more extensively applied in clinical settings.

Our study has several limitations. Firstly, as a single-center retrospective study, it may subject to selection bias. Thus, this novel scoring system requires validation through large-scale multi-centers prospective studies. Secondly, the relatively short follow-up period in our study limit the accuracy of survival predictions beyond 3 years for DLBCL patients. Thirdly, our study primarily focused on the prognostic value of nutritional and immune-inflammatory variables at the time of initial diagnosis. However, the dynamic changes in these variables during anti-tumor treatment and their influence on patient outcomes require further investigation. Lastly, our study did not capture the infection status of patients at initial diagnosis, nor did it assess how the new scoring model impacts the prognosis of DLBCL patients under different infection statuses.

## Data Availability

The original contributions presented in the study are included in the article/Supplementary material, further inquiries can be directed to the corresponding authors.
