# Supplementary material for: Nutritional and immune-inflammatory scoring system for predicting outcomes in newly diagnosed diffuse large B-cell lymphoma patients
Source: Front Nutr. 2025 Jul 28;12:1591508. doi: 10.3389/fnut.2025.1591508 (PMC12336136; doi:10.3389/fnut.2025.1591508)
Supplement: Supplementary file 1 [file Table_1.docx]

**
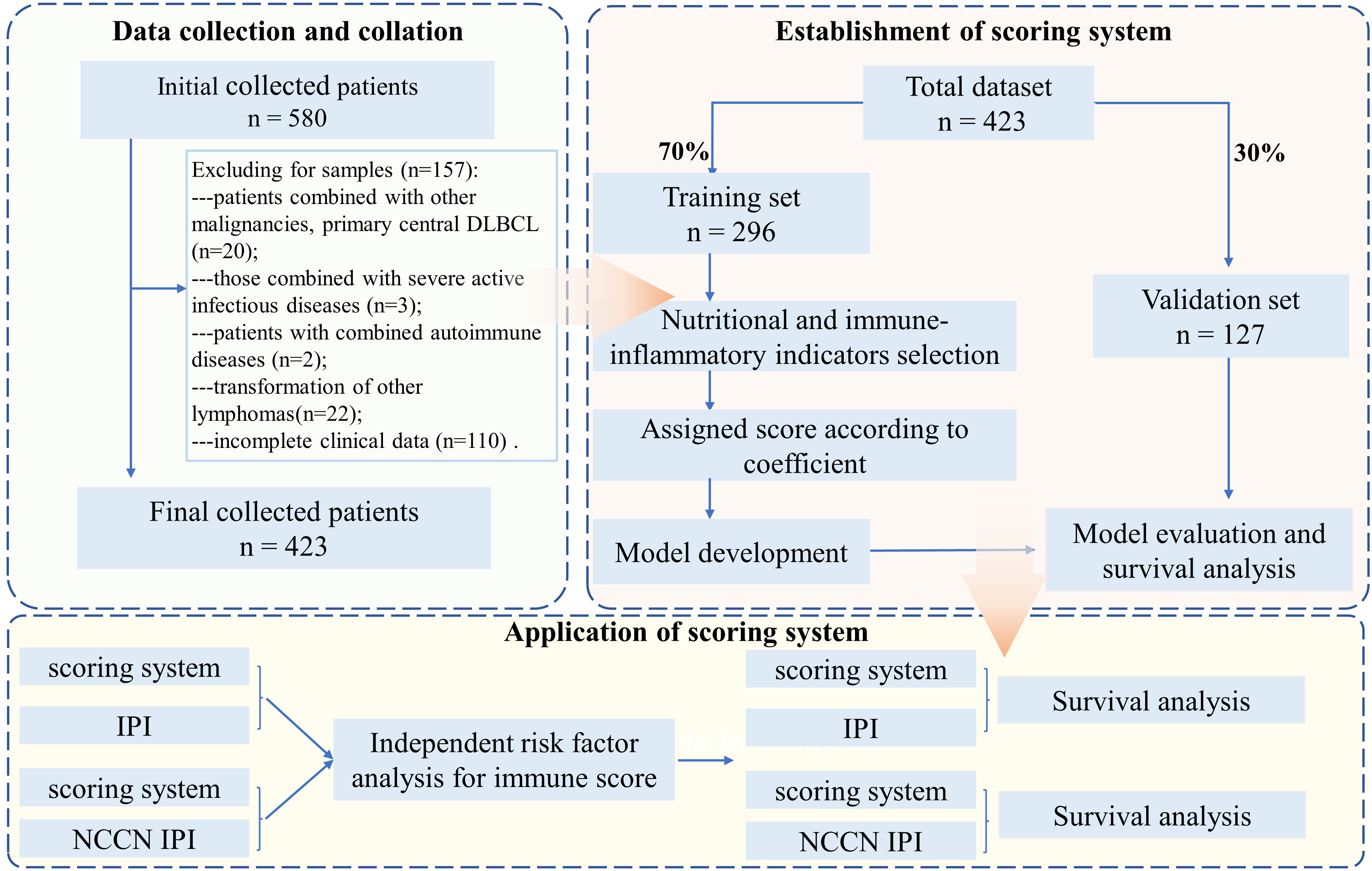
**

**Figure S1. Flow diagram of selecting DLBCL patient and the construction of scoring system**

**Table S1. An Optimal Cut-Off Value of Each Indicator**

| **Indicator** | **Cut-off value** |
| --- | --- |
| Nutritional Risk Screening 2002 (NRS2002) | 3 |
| Geriatric Nutritional Risk Index (GNRI) | 98 |
| Prognostic Nutritional Index (PNI) | 45.85 |
| Systemic immune-inflammation index (SII) | 402.71 |
| Neutrophil to lymphocyte ratio (NLR) | 3.76 |
| Platelet to lymphocyte ratio (PLR) | 153.3 |
| Lactate dehydrogenase to albumin ratio (LAR) | 158.52 |
| β2-MG, mg/L | 4.75 |
| B cells, cells/μL | 116 |
| CD4^+^ T cells, cells/μL | 575 |
| CD8^+^ T cells, cells/μL | 222 |
| CD3^+^ T cells, cells/μL | 857 |


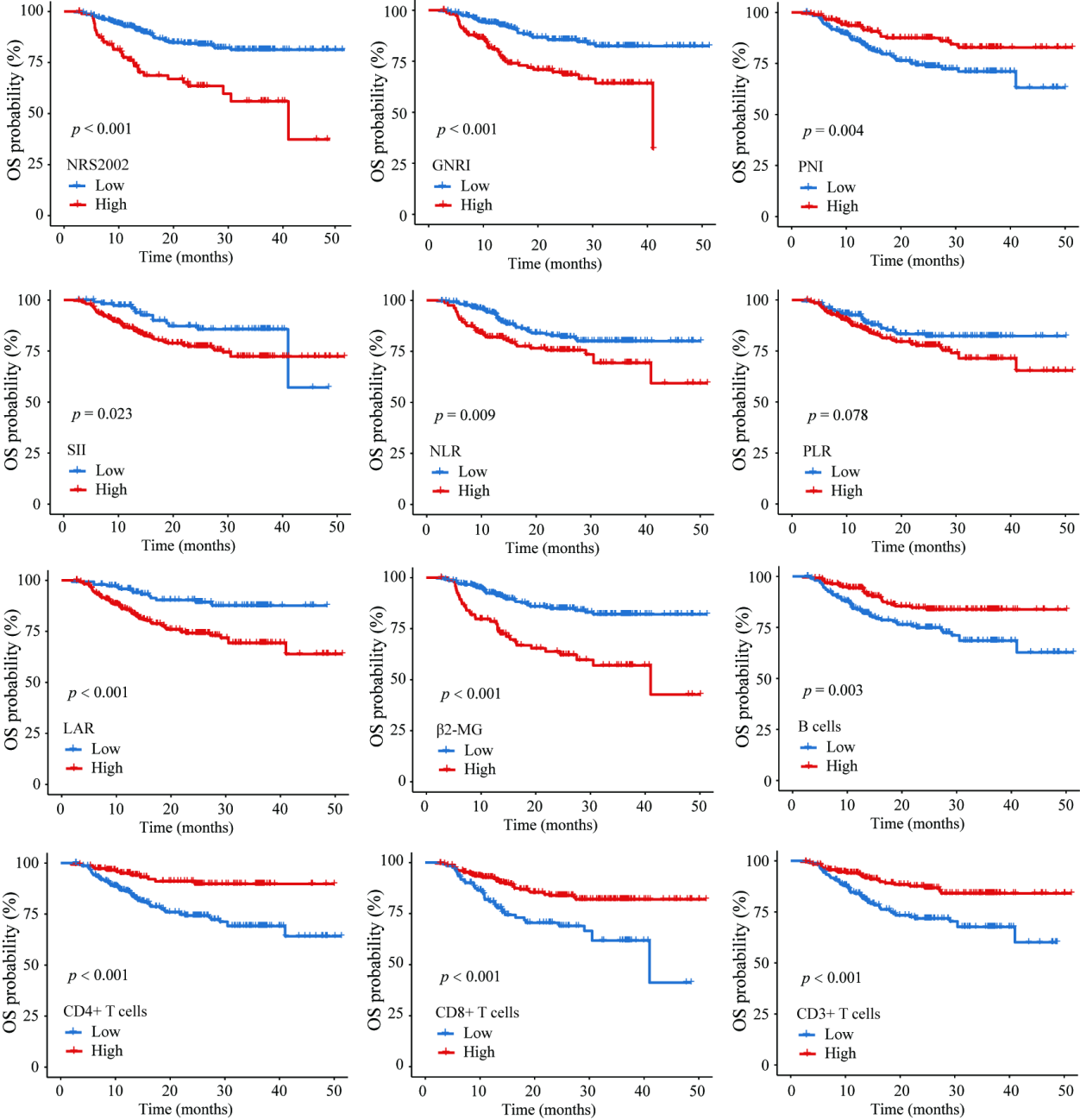


**Figure S2. Kaplan–Meier analysis based on optimal cut-off values of each indicator in DLBCL patients**

Abbreviations: NRS2002, Nutritional Risk Screening 2002; GNRI, Geriatric Nutritional Risk Index; PNI, Prognostic Nutritional Index; SII, systemic immune-inflammation index; NLR, neutrophil-to-lymphocyte ratio; PLR, platelet-to-lymphocyte ratio; LAR, lactic dehydrogenase to albumin ratio; β2-MG, β2-microglobulin.


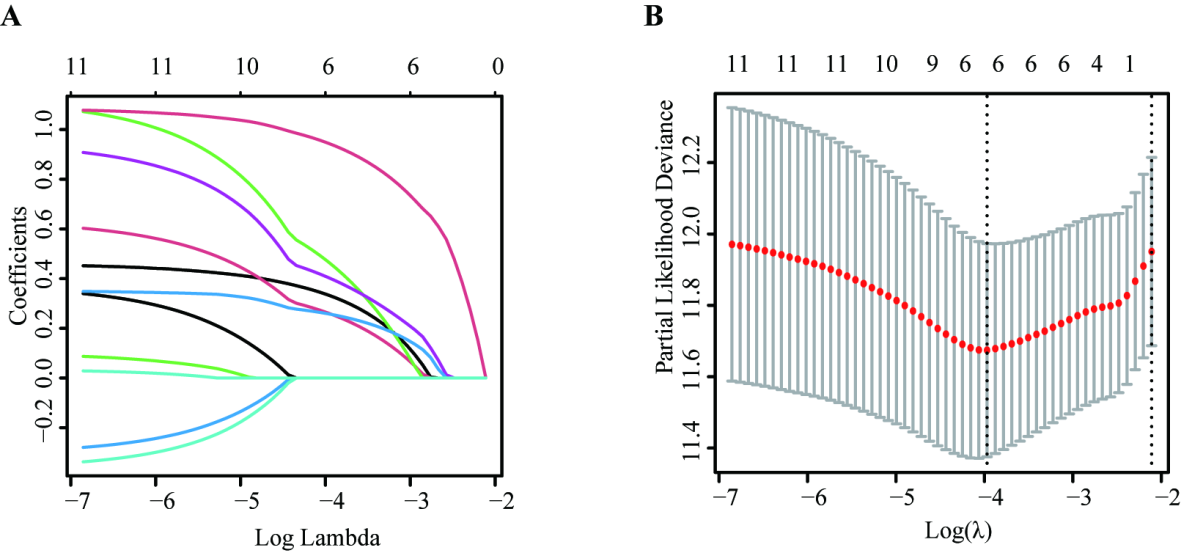


**Figure S3 The least absolute shrinkage and selection operator (LASSO) Cox regression model was used to select the elements to build NII.** (A) The LASSO coefficient profiles of nutritional and immune-inflammatory indicators. (B) Cross-validation error curve for tuning parameter (λ) selection.

**Table S2. VIF and Regression unpenalized Coefficient (β) of Each Index and**

**Corresponding Score**

| **Indicator** | **VIF** | **β (HR=e^β^ )** | **Score** |
| --- | --- | --- | --- |
| NRS2002, ≥ 3 | 1.11 | 0.452 | 1 |
| GNRI, ≤ 98 | 1.15 | 0.392 | 1 |
| SII, ≥ 402.71 | 1.13 | 0.774 | 2 |
| LAR, ≥ 158.52 | 1.22 | 0.579 | 2 |
| β2-MG, ≥ 4.75 mg/L | 1.13 | 1.077 | 3 |
| CD8^+^ cells, < 222 cells/μL | 1.15 | 0.316 | 1 |

Abbreviations: NRS2002, Nutritional Risk Screening 2002; GNRI, Geriatric Nutritional Risk Index; SII, systemic immune-inflammation index; LAR, lactic dehydrogenase to albumin ratio; β2-MG, β2-microglobulin.

**Table S3. The Univariable and Multivariable Analysis for Progression-free Survival in DLBCL Patients**

| Characteristics | Univariable analysis | |  | Multivariable analysis | |
| --- | --- | --- | --- | --- | --- |
|  | HR (95% CI) | *p* value |  | HR (95% CI) | *p* value |
| NII ( <6 vs ≥6) | 3.50 (2.48 - 4.94) | <0.001 |  | 2.44 (1.65 - 3.60) | <0.001 |
| Gender (Male vs Female) | 1.16 (0.82 - 1.64) | 0.410 |  |  |  |
| Age ( >60 vs ≤60) | 1.93 (1.37 - 2.72) | <0.001 |  | 1.60 (1.12 - 2.29) | 0.009 |
| COO classification (non-GCB vs GCB) | 0.75 (0.50 - 1.13) | 0.171 |  | 0.85(0.56 - 1.27) | 0.422 |
| Ann Arbor stage (III/IV vs I/II) | 2.57 (1.68 - 3.92) | <0.001 |  | 1.45 (0.88 - 2.39) | 0.149 |
| ECOG PS (>2 vs ≤ 2) | 1.77 (1.25 - 2.52) | 0.001 |  | 1.34 (0.93 - 1.93) | 0.118 |
| Tumor size ( ≥7.5cm vs <7.5 cm) | 1.35 (0.84 - 2.15) | 0.211 |  |  |  |
| EN site involvement ( ≥ 2 EN site vs < 2 EN site) | 2.04 (1.45 - 2.87) | <0.001 |  | 1.52 (1.03 - 2.24) | 0.033 |
| EBV infection (Yes vs No) | 1.22 (0.62 - 2.40) | 0.566 |  |  |  |
| B symptoms (Yes vs No) | 1.03 (0.70 - 1.51) | 0.878 |  |  |  |
| LDH (>250 U/L vs ≤250 U/L) | 2.15 (1.50 - 3.09) | <0.001 |  | 1.05 (0.68 - 1.62) | 0.838 |
| Tretment regimen (R-CHOP vs other R regimen) | 1.12 (0.80 - 1.59) | 0.504 |  |  |  |
| Tretment cycles (4-5 vs ≥6 ) | 1.21 (0.78 -1.87) | 0.390 |  |  |  |

Abbreviations: NII, nutritional and immune-inflammatory scoring system; COO, the cell of origin; ECOG PS, Eastern Cooperative Oncology Group Performance Status; EN, extranodal; LDH, lactate dehydrogenase; R-CHOP, rituximab, cyclophosphamide, adriamycin, vincristine, and prednisone; other R regimen, R-EPOCH (rituximab, etoposide, doxorubicin, cyclophosphamide, vincristine, prednisone), R-CDOP (rituximab, cyclophosphamide, doxorubicin liposome, vindesine, and prednisone), etc.
